# Do schistosome vaccine trials in mice have an intrinsic flaw that generates spurious protection data?

**DOI:** 10.1186/s13071-016-1369-9

**Published:** 2016-02-17

**Authors:** R. Alan Wilson, Xiao-Hong Li, William Castro-Borges

**Affiliations:** Centre for Immunology and Infection, Department of Biology, University of York, Heslington, York, YO10 5DD UK; National Institute of Parasitic Diseases, Chinese Center for Disease Control and Prevention, Shanghai, 200025 People’s Republic of China; Departamento de Ciências Biológicas, Universidade Federal de Ouro Preto, Campus Morro do Cruzeiro, Ouro Preto, Minas Gerais Brasil

**Keywords:** *Schistosoma mansoni*, *Schistosoma japonicum*, Radiation attenuated vaccine, Antigen vaccine, Mouse, Hamster, Primate, Intravascular migration, Maturation, Pulmonary capillary, Inflammation

## Abstract

The laboratory mouse has been widely used to test the efficacy of schistosome vaccines and a long list of candidates has emerged from this work, many of them abundant internal proteins. These antigens do not have an additive effect when co-administered, or delivered as SWAP homogenate, a quarter of which comprises multiple candidates; the observed protection has an apparent ceiling of 40–50 %. We contend that the low level of maturation of penetrating cercariae (~32 % for *Schistosoma mansoni*) is a major limitation of the model since 68/100 parasites fail to mature in naïve mice due to natural causes. The pulmonary capillary bed is the obstacle encountered by schistosomula en route to the portal system. The fragility of pulmonary capillaries and their susceptibility to a cytokine-induced vascular leak syndrome have been documented. During lung transit schistosomula burst into the alveolar spaces, and possess only a limited capacity to re-enter tissues. The acquired immunity elicited by the radiation-attenuated (RA) cercarial vaccine relies on a pulmonary inflammatory response, involving cytokines such as IFNγ and TNFα, to deflect additional parasites into the alveoli. A principal difference between antigen vaccine protocols and the RA vaccine is the short interval between the last antigen boost and cercarial challenge of mice (often two weeks). Thus, after antigen vaccination, challenge parasites will reach the lungs when both activated T cells and cytokine levels are maximal in the circulation. We propose that “protection” in this situation is the result of physiological effects on the pulmonary blood vessels, increasing the proportion of parasites that enter the alveoli. This hypothesis will explain why internal antigens, which are unlikely to interact with the immune response in a living schistosomulum, plus a variety of heterologous proteins, can reduce the level of maturation in a non-antigen-specific way. These proteins are “successful” precisely because they have not been selected for immunological silence. The same arguments apply to vaccine experiments with *S. japonicum* in the mouse model; this schistosome species seems a more robust parasite, even harder to eliminate by acquired immune responses. We propose a number of ways in which our conclusions may be tested.

**Electronic supplementary material:**

The online version of this article (doi:10.1186/s13071-016-1369-9) contains supplementary material, which is available to authorized users.

## Background

The question posed in the title of this review was prompted by two recent sets of observations. Firstly, in 2014, publications from three Brasilian groups reported protection against schistosome challenge after vaccination of mice using what are unquestionably intracellular proteins. The four proteins were Syntenin [1], Dynein light chains DLC12 and DLC13 [2], and Y box protein [3]. Secondly, we undertook a quantitative shotgun proteomic analysis of the ubiquitous antigen preparation SWAP, the soluble cytosolic extract of adult worms [4]. We found that about one quarter of the protein mass comprised putative vaccine candidates identified by other researchers. In this situation why does SWAP, packed with multiple vaccine candidates, not have a dramatic protective effect? Instead it performs no better than individual candidates administered alone, and sometimes worse. Do these antigens all trigger the same mechanism, which has a ceiling of approximately 40–50 % protection, whether one or many are used?

In this review we ask whether the protective outcome of antigen vaccination is an artefact of the mouse model and not a measure of acquired immunity. This is not the same as saying that there are no protective antigens but simply that the mouse model may not be capable of discriminating between acquired immunity induced by antigens and an effect of vaccination on host physiology that diminishes schistosome maturation in some non-specific way.

## The mouse as an animal model for schistosomes

Whilst the laboratory mouse is a convenient host for schistosomes, used in hundreds of vaccine testing studies over the last 40 years, it does have a major drawback. It is described as a permissive host but in reality the actual numbers of penetrant *Schistosoma mansoni* parasites that migrate from the skin to the portal system and mature into adult worms is quite low. The statistic can be obtained by counting the percentage of challenge control cercariae that reach maturity in mice exposed to the radiation-attenuated (RA) cercarial vaccine but receiving no adjuvant or other treatment. In a sample of 26 experiments from the York group over a decade, using the Puerto Rican isolate, the maturation in C57Bl/6 mice was 32.3 % [5–9]. The maturation of cercariae of the same isolate in CBA mice in seven experiments by the London School of Hygiene group was an almost identical 32.5 % [10]. The precise values may differ with isolate and mouse strain but the stark fact from these data is that of 100 cercariae penetrating the skin of a *naive* mouse, 68 will fail to reach and mature in the site of parasitisation, the portal tract. Vaccine-induced protection is defined as the reduction in burden between vaccinated and control groups and the low maturation in mice is dealt with by using the formula: % Protection = (Control burden - Test burden)/Control burden × 100. For example with a ~40 % level of protection, in the control group 68 parasites will fail to mature, while vaccination of the test group eliminates only a further 13 parasites. Thus, of the 81 parasites that did not mature in the test mouse, the vaccine treatment only accounted for (13/81 × 100 =) ~16 %; the rest died of natural causes. In truth, the way of calculating protection in the mouse very effectively disguises only a small achievement in worm elimination.

A larger number of penetrant *S. mansoni* cercariae mature in the golden (Syrian) hamster with a mean of 56 % recorded in eight experiments [11–14], and values as high as 76 % in single experiments [15]. In non-human primates, the hosts evolutionarily closest to humans, there is comparable data available from challenge control animals in vaccine experiments with RA cercariae. A maturation of 82 % was recorded in a single vervet experiment (*Cercopithecus aethiops* [16]) and a mean of 80.5 % in three baboon experiments (*Papio anubis* [17]). In contrast, the laboratory rat presents an even greater obstacle to migration than the mouse, with 22–27 % of juvenile worms detected in the liver between days 11 and 21 [18]. We conclude that the laboratory mouse, viewed solely in the context of parasite maturation, does not seem the best choice for vaccine experiments, and rats are even worse.

## Exactly what are the “natural causes” that limit maturation?

It is important to establish at the outset that the 68 % of non-maturing *S. mansoni* penetrants are not eliminated by immunological processes as their protracted migration and slow development (~5 weeks) allow ample time for immune intervention. The best evidence is provided by experiments in which mice were exposed to whole body irradiation a few days before infection. The treatment severely depresses immunological responsiveness without administration of chemicals or alteration of genotype. Most parameters decline by ~90 %, yet parasite migration and maturation are not enhanced [19–21]. This strongly supports a physical explanation for the low maturation.

**Fig 1:**
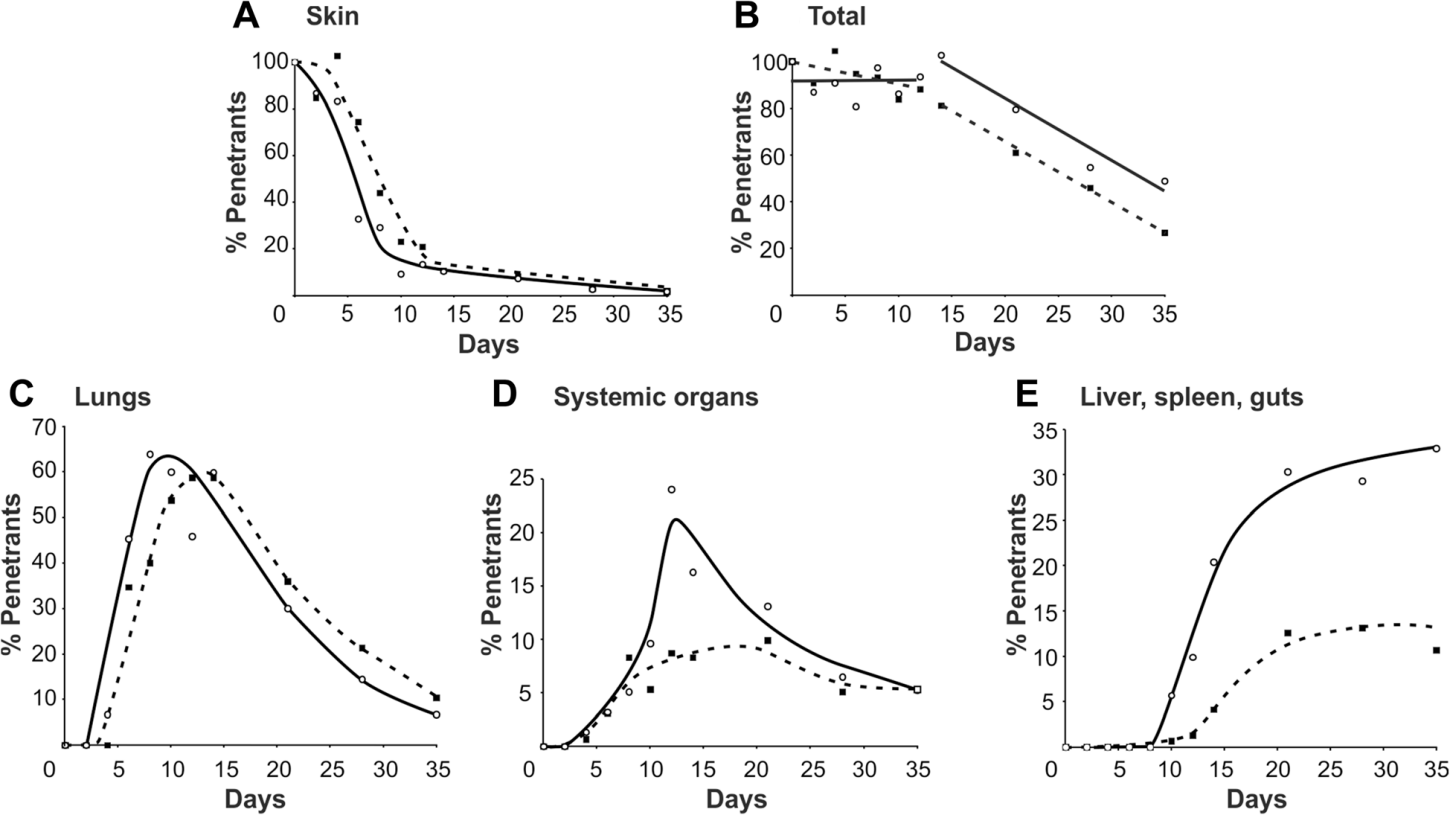
Balance sheet showing the profile of schistosome migration through all organs of the mouse. The numbers of schistosomula detected by compressed organ autoradiography after exposure of mice to ^*75*^Se-Methionine-labelled cercariae (reproduced with permission from [22]). Open circles and solid lines are normal mice, closed circles and dashed lines are mice vaccinated five weeks previously with 500 RA cercariae. **a** and **c** The vast majority of schistosomula leave the skin and travel to the lungs. **d **and **e** The impact of vaccination is revealed by the reduced parasite numbers detected in the systemic organs and accumulating in the portal system, compared to normal mice. **b **The decline in parasite numbers only begins from day 15 at which time < 10% remain in the skin. The lungs are the principal obstacle to maturation in normal mice, and more so in vaccinated animals

As a result of autoradiographic tracking experiments on ^75^Seleno-methionine-labelled parasites we now have explicit data on the route taken by schistosomula from the skin to the portal system, the kinetics of the process, and their fate along the way (Fig. 1; reviewed in [22, 23]). The idea that most non-maturing penetrants died in the skin was quickly dispelled and the mincing and incubation of tissues to extract the larval parasite burden was discredited as a quantitative technique [24]. A careful balance sheet of parasites in all mouse organs up to 35 days post-infection revealed that the vast majority exited the skin (Fig. 1a) and transited to the lungs, where peak numbers of > 60 % penetrants were detected (Fig. 1c) [25]. Note that the first larvae arriving in the lungs pass through the vascular beds and exit before the last larvae arrive from the skin, so the maximum numbers in the lungs never equal the skin total. Schistosomula begin to leave the lungs around 5 days post-infection *via* the venous circulation to the left side of the heart and are distributed in arterial blood to all organs of the body (Fig. 1d). The fractional distribution of cardiac output in the mouse is approximately 0.33:0.66 splanchnic:systemic organs. The one third of schistosomula entering the splanchnic arteries (coeliac, superior and inferior mesenteric) pass through the gastrointestinal capillary beds to reach the portal system (Fig. 1e); the remaining two thirds negotiate the capillary beds of systemic organs to return to the lungs. The principal feature revealed by the balance sheet is that no parasites are eliminated before 15 days post-infection (Fig. 1b), at which time < 10 % can be detected in the skin; this is the clearest possible demonstration that parasite death in the skin is minimal. At 15 days the population is distributed around all organs of the mouse body, with the largest proportion in the lungs. The question is ‘what happens from 15 days onwards’?

**Fig 2:**
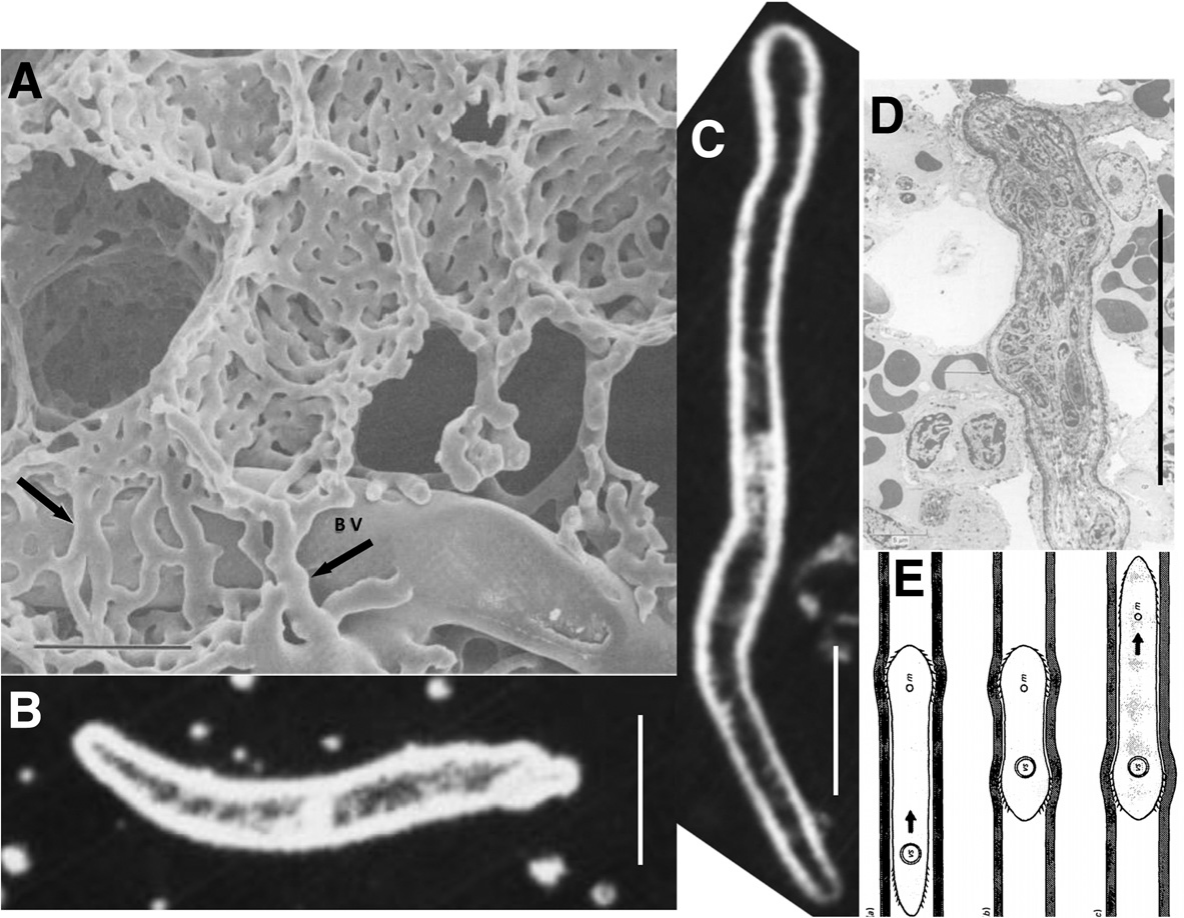
The morphology of pulmonary migration. **a** Cast showing the complex meshwork of short capillaries that surround each alveolar space, in contrast to more normal capillaries at the periphery (*arrow*). BV, blood vessel. The mean diameter of mouse alveoli ranges from 36 to 45 μm, depending on strain while the diameter of an alveolar capillary is ~ 6 μm. Image from Guntheroth et al. [35] with permission. **b** Dark-field image of a Day 2 schistosomulum prior to exit from the skin *via* a blood vessel. **c** Dark-field image of a fully extended Day 6 lung schistosomulum, approximately 400 μm in length. When crossing the vascular bed, the parasite body must extend through the capillary labyrinth of several alveoli (**b** and **c** from [29]). **d** Transmission electron micrograph of a part of a lung schistosomulum lying within the lumen of an alveolar capillary. The tight squeeze is confirmed by the bulge in the body where it lies adjacent to another capillary (arrowed) (from [30]). **e** Diagrammatic representation of the way that a schistosomulum uses its anterior and posterior body spines to move along the capillary lumen, alternately anchoring one set and then the other (from [36]). Scale-bars in **a**–**c**: 50 μm

When migrating schistosomula, recovered from the pulmonary vasculature of donor mice, are injected *via* a vein back to the lungs of naïve recipients, ~50 % reach maturity [26, 27]. However, > 80 % of the same batch, injected into the portal vessels, are recovered as mature worms four weeks later. Such experiments confirm that the mouse lungs are the real barrier on the migratory route. The reason why less than 100 % of injected schistosomula develop to maturity in the portal tract is that as many as 25–30 % actually negotiate the hepatic sinusoids back to the venous circulation and lungs [26]. They must then traverse the lung obstacle again, as must those schistosomula returning from systemic organs. The magnitude of the lung barrier is underlined by experiments to determine the kinetics of larval migration through different vascular beds [22, 23, 28]. The mean transit time of day 7 schistosomula through the lungs is 30–35 h. This contrasts with values of 16 h for systemic organs and 6.5 h for intestinal capillary beds [28] (estimates from an independent experiment with fewer time points gave values of 11.7 and 9.8 h, respectively [26].) Clearly negotiating the lung capillaries is hard work. It must be emphasised that schistosomula leaving the skin are short and stubby (100 to 190 μm long, Fig. 2b; [29]) and completely occlude pre-capillary arterioles of the lungs 10 to 20 μm in diameter, when they arrive [30]. They must then undergo a phase of development, becoming longer (> 400 μm), thinner (~8 μm) and losing mid body spines (Fig. 2c; [29, 30]) to facilitate transit along capillaries only 7 μm wide. For the earliest arrivals, the first transit through the lungs may take 78 h. In addition, the percentage maturation for Day 3 schistosomula delivered i.v. to the lungs is 30 % and for Day 4 schistosomula 41 %, compared with ~50 % for Day 7 larvae [15]. These data suggest that the first transit is the most difficult and protracted; indeed, it is quite possible that some schistosomula arriving from the skin never progress further [15]. Conversely, the percentage maturation of Day 12 and Day 17 schistosomula delivered to the lungs i.v. is not significantly different from that of Day 7 larvae [27]. We should therefore consider the Day 7 lung schistosomulum as fully adapted to intravascular migration [29].

A combination of light and electron microscopy revealed that at day 7 post-infection all lung schistosomula were located in blood vessels of naïve mice [31]. At the next sampling time, day 11, a proportion of them were entirely or partially intra-alveolar, and this reached 80 % at day 20 and later. No cellular reactions were evident around intravascular parasites but at later sampling times alveolar parasites were associated with large inflammatory foci, probably elicited as a response to non-specific tissue damage when they burst out. In spite of this inflammation, no damage to the schistosomula was observed. These observations were confirmed in an autoradiographic study which concluded that the alveolar larvae were unharmed and were likely to be coughed up, swallowed and digested [32]. Administration of schistosomula *via* the trachea to the pulmonary airspace revealed that a small number (~15 %) were able to re-enter blood vessels, migrate and mature in the liver, but this proportion diminished as the parasites aged [27]. Exit into alveoli appears to occur accidentally as parasites attempt to traverse pulmonary blood vessels, and once there it becomes difficult to continue migration.

We can estimate the proportion of schistosomula becoming trapped and diverted to the airspaces on each circuit using a simple spreadsheet calculation (Additional file 1: Table S1A). An average *P* value of 0.36 for a schistosomulum getting stuck on each passage through the lung vascular bed (including the first) creates a realistic migration profile. Only a few passages around the circulation, each taking about two days, are sufficient to generate the cumulative portal population. The remaining parasites blunder into the airspaces of the lungs or get stuck in other locations (Fig. 1). This physical explanation of worm demise, with the average *P* value of 0.36, accounts almost precisely for the ~32 % maturation in naïve mice. However, the maturation data for Day 3 and Day 4 schistosomula indicate that they have *P* values for getting stuck of *P* = 0.415 and 0.28, respectively, while the 50 % maturation of day 7 (or older) larvae delivered i.v. to the lungs is achieved by *P* = 0.203 (Additional file 1: Table S1B). So what feature of the lungs, especially mouse lungs, presents a special obstacle to onward migration?

The first and most obvious aspect is that the pulmonary capillaries have very thin walls separated from the airspaces only by alveolar basement membrane and epithelium. The total thickness of the blood-gas barrier is ~0.3 μm with the alveolar epithelial cell in some parts having a thickness of 0.1 μm [33]. Thus the pulmonary capillary is only separated from the alveolar space by a layer of extracellular matrix and an epithelial cytoplasm, each 0.1 μm thick. No other capillary in the body is protected by such a thin layer of tissue, making the pulmonary capillaries fragile and vulnerable to failure [33]. The mechanical behaviour of alveolar capillaries is determined by the extracellular matrix layer, with type IV collagen the most important constituent determining the strength of the blood-gas barrier. The combined thickness of the three components of the blood-gas barrier correlates with the experimental pressures required to damage to the pulmonary capillary wall in different species, the larger the animal, the thicker the blood-gas barrier (horse > dog > rabbit) [34]. Such measurements are not available for the mouse but it is plausible that the alveolar capillaries of this species may be particularly fragile due to its small size. Indeed, it is easy to appreciate how the vigorous and rhythmic extensions and contractions displayed by elongating schistosomula [29], especially the younger larvae, could provide the motive power to rupture alveolar capillaries in the mouse lung.

A second difference is the nature of the pulmonary capillaries themselves. Those enveloping the alveoli are very short (~10 μm) forming a maze of vessels [35] through which the schistosomulum must crawl (Fig. 2a). This contrasts with the network of longer bifurcating tubules that comprise the capillary bed of other organs and non-alveolar parts of the lungs (Fig. 2a, arrowed). Juxtaposing an image of an extended Day 6 lung schistosomulum (Fig. 2c) next to that of the alveolar capillaries brings the task it faces in traversing the pulmonary vascular bed into sharp focus. Unsurprisingly it must take a very convoluted path through the maze of short tubes with many options, as revealed by in situ electron microscopy (Fig. 2d; [30]). Adding in the fact that it progresses along vessels in inchworm fashion using its anterior and posterior spines (Fig. 2e; [36]) everything converges to make pulmonary vascular transit a difficult proposition, with a significant probability of bursting into an alveolus.

The lungs are also subject to another potentially relevant phenomenon, the vascular leak syndrome (VLS). VLS was first reported following cytokine administration (Interleukin 2) to patients to treat certain cancers [37] and has been explored using the mouse as a model. It is characterised by leakage of vascular fluids into tissues, accompanied by hypotension; in the lungs this results in pulmonary oedema. There is a general consensus that IL-2-induced VLS is caused by secondary release of inflammatory cytokines such as IFNγ and TNFα [38, 39], which may in turn increase production of vascular mediators such a nitric oxide (NO). It has even been suggested that VLS may be accompanied by modifications in the extracellular matrix [37], which could weaken the alveolar blood-air barrier to increase capillary fragility. The relevance of VLS to schistosome vaccines is that its causative agents are precisely those cytokines and mediators involved in the protective effector mechanism elicited by the RA vaccine (see below).

## The radiation-attenuated (RA) cercarial vaccine in mice

The best studied model of acquired immunity to schistosomes is that induced by exposure of rodents and primates to a dose of RA cercariae. We have a wealth of immunological and parasitological information about the actions of attenuated parasites in generating protection and the fate of a normal challenge in vaccinated hosts (reviewed in [40–42]). In mice, optimally attenuated parasites undergo a truncated migration as far as the lungs, priming the immune response *via* skin-draining lymph nodes. These attenuated larvae also “arm” the lungs by stimulating recruitment of effector T cells to the interstitial spaces and airways [43]. Multiple vaccinations have an additive effect and almost sterile immunity can be achieved by co-administration of attenuated larvae and the cytokine IL-12 [44, 45]. However, we now know that subcutaneous administration of IL-12 can produce adverse systemic effects [46]. Initial priming has a strong Th1 component but subsequent exposures to RA cercariae amplify Th2 cells and enhance antibody involvement. A striking feature is that a period of five weeks is left after application of the attenuated parasites, before challenge with normal cercariae. This allows any non-specific inflammatory events to subside before challenge parasite entry; vaccination and challenge are normally performed on separate sites such the abdominal skin and the tail, to avoid residual inflammation left by the initial exposure.

**Fig 3:**
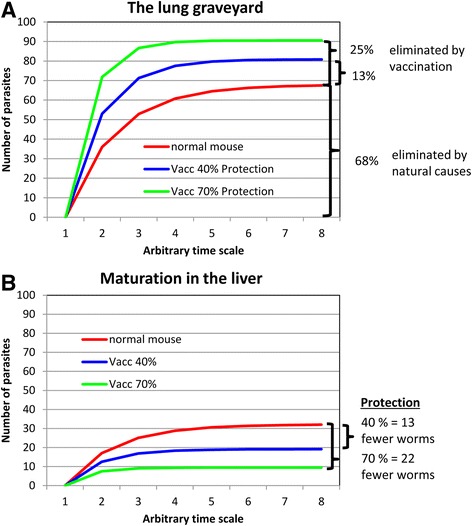
Diagrammatic representation of the fate of migrating schistosomula in normal and vaccinated mice. **a** Cumulative parasite deaths in the lungs. **b** Cumulative parasite survival and development in the portal system. The diagrams are based on the spreadsheet calculation in Additional file 1: Table S1. For simplicity, the model assumes that parasites end their migration either in the lungs or the portal system. After an initial elongation phase of about three days in the lungs, the time-base of a circuit around the body in the bloodstream is approximately 48 h. An average *P* = 0.36 of getting stuck in the lungs of a naïve mouse on each passage produces an adult worm portal population equal to 32 % of penetrating cercariae (red line). A “protection” of 40 % induced by a vaccine needs to increase the *P* of getting stuck to 0.53, with 13 more parasites trapped (blue line). A “protection” of 70 % needs a *P* of 0.72, with 22 more worms trapped. This is still only one quarter of the total losses

What happens to challenge parasites when they penetrate the skin of previously vaccinated mice? They are a little slower to leave but the population still travels to the lungs where the discrepancy with migration in a naïve mouse becomes apparent (Fig. 1). Fewer larvae transit the lungs to reach the systemic and splanchnic organs, as evidenced by the lower peak numbers in the first (Fig. 1d) and the slower build up to a lower plateau in the second (Fig. 1e). Total numbers detected begin to decline a little earlier in the vaccinated hosts and proceed to a lower end point but the rates of parasite elimination are identical (Fig. 1b). Even without knowledge of immunological events or mechanisms, it is clear that the major site of parasite loss is the lungs. This organ, difficult to negotiate in naïve mice, becomes more so in vaccinated animals. Increasing the chance of getting stuck on each circuit from *P* = 0.36 to *P* = 0.53 predicts the 40 % reduction in worm burden (Additional file 1: Table S1), as an additional 13 parasites fail to mature beyond the 68 in naïve animals (Fig. 3). The corresponding values for a 70 % reduction in burden are *P* = 0.72 with an additional 22 parasites prevented from maturing, still less than a quarter of the total.

What is the evidence that the protection induced by the RA vaccine has an immunological basis? Adoptive transfer of the immunity elicited by the RA vaccine has been achieved using a parabiotic union between mice [47]. Subsequent parabiotic experiments where the naïve and vaccinated partners were separated before challenge demonstrated two key facts about the model [9]. Firstly, recruitment of effector cells to the lungs was a crucial pre-arming component of the protective response. Secondly, an anamnestic response was elicited by percutaneous challenge and could act against migrating larvae in the lungs. However, when the challenge was provided by day seven schistosomula administered directly to the lung vasculature, no protection was evident. Passive transfer of protection with homologous serum from vaccinated mice proved difficult to demonstrate [48]. However, it was eventually achieved using serum from multiply vaccinated mice [49], especially high-titre serum from IFNγR^-/-^ knock-donors [50]; in both studies the serum could be administered as late as day 4 or 7 post-challenge, revealing that the target was the lung, not the skin schistosomulum. To our knowledge, the passive or adoptive transfer of protection to naïve mice has not been achieved using serum or cells respectively, from any donors reportedly exhibiting antigen-induced acquired immunity.

How are the extra challenge schistosomula arrested in the lungs? A focal inflammatory response develops to each larva over several days [51]. The mass of cells around the parasite hinders onward migration and increases the probability of deflection into an alveolus. IFNγ is a key cytokine [52, 53] and production of TNFα also appears to be central to protection; mice lacking the TNF receptor 1 (TNFRI^-/-^) were not protected by exposure to the RA vaccine [8]. However, pulmonary inflammation alone is not sufficient since the cell infiltration around migrating larvae in the lungs of IFNγR^-/-^ and TNFRI^-/-^ mice is actually greater, but is ineffective at increasing the proportion of schistosomula deflected into the alveoli; this implies that there are other downstream mediators. Strikingly, the protection does not involve direct immune damage to schistosomula. Almost no evidence of cytological damage inflicted by the enveloping inflammation was observed in trapped challenge larvae in situ [31] and lung schistosomula were not susceptible to ADCC mechanisms *in vitro* [54]. Most telling, transfer experiments with challenge schistosomula trapped in the lungs (which would not mature if left in situ) showed that they could migrate and develop normally if introduced into the pulmonary or portal circulation of naïve animals [27]. This durability is a testament to the anti-oxidant capabilities of migrating larvae [55]. In summary, the inflammatory effector mechanism of the RA vaccine apparently alters the pulmonary environment to decrease the probability that a schistosomulum can successfully traverse the vascular beds, but it does not cause it direct harm.

**Fig 4:**
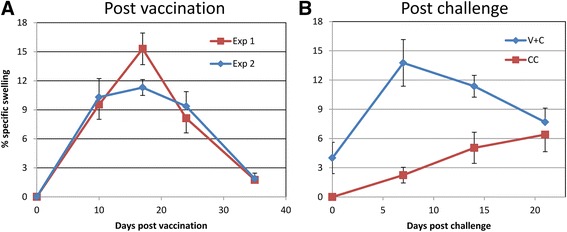
Activated T cells in the circulation detected by footpad Delayed-Type Hypersensitivity (DTH) assay. **a** Reactivity after vaccination with 500 RA cercariae. Two identical experiments are plotted showing the Th1 cells reach a peak at approximately 17 days and have declined almost to background levels before challenge at 35 days. **b** Reactivity of vaccinated mice (V + C) and naïve controls (CC) challenged at 5 weeks with 200 normal cercariae. A sharp increase in circulating T cells is observed 7 days after challenge of the vaccinated animals, coincident with peak schistosomula numbers in the lungs, and then a gradual decline. The reactivity of the control mice is muted in comparison, both with respect to the previously vaccinated animals and to the naïve mice in part A exposed to RA cercariae. The gradually rising DTH response of the CC group up to day 20 is most likely a response to worm vomitus released by the blood feeding worms accumulating in the liver. Replotted from [57]

Although the effector mechanism in RA vaccinated mice operates in the lungs, the effector T cells were generated in the lymph nodes draining the skin vaccination site. They exited *via* the efferent lymphatics to join the circulation from where they were available for recruitment to the lungs by the attenuated vaccinating schistosomula [56]. Such circulating effector T cells can also be recruited to other sites like the footpad or pinna by an inflammatory stimulus and this forms the basis of a delayed-type hypersensitivity (DTH) assay to estimate their level in the circulation. In mice exposed to the RA vaccine, DTH T cells are high by ten days post-exposure, peak at day 17, are in decline by day 21 and almost down to background by day 35 when the cercarial challenge is administered (Fig. 4 [57]). Exposure to the challenge rapidly stimulates a recall response with a surge of DTH T cells into the circulation, peaking at day 7, declining by day 14 and well towards background by day 21. These data provide the clear justification for the five-week interval between vaccination and challenge, and also key evidence for an anamnestic response to challenge larvae. The same kind of peripheral responsiveness has been observed in chimpanzees exposed to repeated doses of the RA vaccine. There was a progressively increasing reactivity of peripheral blood mononuclear cells to schistosome antigens with each successive vaccination; reactive cells were still circulating four weeks after challenge [58].

## Vaccine antigen testing in the mouse

### How does it differ from RA vaccine experiments?

In testing of vaccine antigens in the mouse a common design is employed. A recombinant protein, less frequently a parasite fraction or a purified protein, is formulated with an adjuvant for subcutaneous or intraperitoneal administration. Freund’s complete adjuvant is a frequent choice for priming followed by two doses of Freund’s incomplete adjuvant as the booster, spaced two or three weeks apart. Mice that will serve as challenge controls receive the adjuvant alone formulated with saline; these are not equivalent. An irrelevant antigen of similar immunogenicity would be a better control; very seldom is a no-adjuvant group included. (Vaccination with DNA constructs follows a similar pattern, but without extraneous adjuvants). The mice are all challenged with the same pool of cercariae at an interval after the last boost, and then perfused 5–8 weeks later to recover and count adult worms.

A major, and we suggest significant, difference from the RA vaccine is the much shorter interval between the last boost and the day of challenge. The preferred time appears to be around 14–15 days but it may be as short as 10 days and less commonly three or occasionally four weeks. Seldom is the interval five weeks as is standard with the RA vaccine in mice, and we can find few instances where it is longer than that. Recent experiments with two tegument vaccine candidates appear to support our contention. When C57Bl/6 mice were vaccinated with Sm22.6 and challenged 15 days after the last boost, they showed a mean 34.5 % protection [59]. In contrast, when the antigen was administered to Balb/c mice that were challenged 30 days after the last boost, the protection elicited was 0 and 18 % [60]. A similar pattern was observed with Sm29 where 51 % protection was observed after a challenge of C57Bl/6 mice at 15 days after the last boost [61] and 0 % protection after a challenge of Balb/c mice at 30 days [60]. Of course it can be argued that mouse strain was the key determinant of protection, not the interval between boost and challenge, a point that can only be resolved by further experiments.

A key feature of our hypothesis is that the short time interval between boost and challenge may provide an explanation for the apparent immunity elicited by some very unlikely vaccine candidates. At the end of the antigen vaccine schedule, is the level of inflammatory cytokines in the circulation sufficient alone to modify pulmonary vascular physiology? Alternatively, do migrating challenge schistosomula elicit an inflammatory stimulus in the lungs that recruits vaccine-activated cells from the periphery, unrelated to their own antigen specificity? Remember that only a small additional number of larvae, above and beyond those that will not make it anyway, need to be arrested by the lung obstacle for 40 or 50 % protection to be generated. It is not 40 or 50 out of 100 penetrants, it is 13 to 16 extra larvae. We suggest that only a small additional effect on the pulmonary capillaries would be needed to impede this extra fraction of schistosomula.

### Are the vaccine antigens plausible candidates?

Over the last three decades quite a long list of schistosome candidates has been put forward, primarily based on the results of protection experiments in mice. The candidates divide into two categories, internal and surface exposed/secreted. The first is larger and well represented in SWAP [4]. It includes TPI, GST, Sm14 fatty acid binding protein (FABP), Aldolase, GAPDH, Calponin, Sm20.8, Sm22.6, paramyosin, myosin heavy chain, 14-4-3 chaperone, and stomatin. The shorter list of exposed proteins, present in small amounts in SWAP, includes: the tetraspanins Sm23 and TSP-2, tegument Sm29 and calpain, and gut-secreted Cathepsin B.

The list encompasses the six WHO candidates that have acquired almost mythical status. Two of them are localised in muscle (paramyosin and myosin heavy chain) and three are cytosolic (TPI, GST, FABP). They did not perform well in independent murine trials but acquired a life of their own such that today they can still be described as the most promising candidates [62]. A goal of 40 % protection for a usable vaccine was set by WHO in the early 1990s so it is evident that, in spite of much work with different adjuvants and live vectors over the intervening 20+ years, a real and perhaps insuperable obstacle to improvement remains. Multiple exposures of mice to irradiated parasites drive protection towards 100 %, whereas co-administration of two vaccine antigens [63, 64] or multiple epitope constructs [65, 66] do not markedly increment protection. The real test in this respect is the administration of SWAP with its multiplicity of candidates, where protection seldom reaches or exceeds 50 % [67, 68]. There has always been a conceptual problem with the cytosolic and cytoskeletal constituents as vaccine antigens. How could an immune effector mechanism “recognise” these internal proteins in a live parasite and interact with them to cause its demise? It is a question that has never received a satisfactory answer from the internal candidate enthusiasts.

### Live BCG as an adjuvant

For one candidate, paramyosin, there a comprehensive body of relevant immunological data. However, the purified protein was only effective when administered with live BCG as an adjuvant. The production of IFNγ and the activation of macrophages were shown to be key components of the model [69] so the parallels with the RA vaccine are obvious. Furthermore, strains with a tendency to generate Th1 type responses were more readily protected than e.g., Balb/c mice, or P strain mice with a defect in macrophage activation [70]. Parasite tracking experiments were mentioned as ‘in progress’ [71] but the site of challenge elimination was never identified. It is noteworthy that both mouse monoclonal and rabbit polyclonal anti-paramyosin antibodies failed to confer any protection when administered -1 and +5 days after infection [72]. A complicating factor in the paramyosin experiments was that i.v. administration of BCG was already known to reduce worm burden non-specifically by generating pulmonary inflammation to interfere with migration [73]. In the paramyosin experiments the BCG was given intradermally (i.d.) with the assumption that it would not enter the circulation and be disseminated, although this does seem not to have been formally tested. However, i.d. administration of BCG, with subsequent detection in internal tissues does occur [74] so it may not be possible to disentangle vaccine antigen and BCG effects, making pulmonary involvement more plausible.

### What can we deduce from the properties of the vaccine candidates?

It is a reasonable assumption that the closer the identity of a schistosome protein to its nearest ortholog in the murine host, the lower the probability that a unique epitope will exist. The candidates range in percentage homology between 29 and 74 % (mean 50 %; Table 1). Bepipred (http://tools.immuneepitope.org/bcell/), the B cell epitope predictor indicates that the larger schistosome proteins have higher numbers of predicted epitopes, with myosin, paramyosin and calpain the winners. The likely immunogenicity of each candidate can be gauged using a simple formula where the number of epitopes is divided by the proportional identity of sequence times the Mw in kDa. On that basis, the leaders are GST-28, Sm20.8 EF hand Ca^2+^ binding protein, Sm14 FABP and calpain (mean 36 % homology). The least immunogenic are Enolase, 14-3-3, Stomatin and Aldolase (mean 66 % homology). (Indeed enolase is only included in the list to make the point that when we cloned and expressed it for vaccine experiments we could achieve no protection at all.) Our contention is that in the mouse model pure immunogenicity is likely to be the best predictor of an effect, simply because it will provide the strongest stimulation to the immune system.

**Table 1:** Molecular properties of vaccine candidates

GeneDB	Annotation	% id Mus	Mouse orthologue	Accession #	# epitopes > 4AA^a^	MW (kDa)	Index
Smp_054160	GST28	32	prostaglandin D synthase	NP_062328.3	8	23.8	1.05
Smp_086530	Sm20.8	29	non-muscle alpha-actinin 4	ABC66068.1	5	20.8	0.83
Smp_095360	Sm14	41	fatty acid-binding protein	NP_067247.1	5	14.8	0.82
Smp_214190	Calpain	40	calpain Lp82	AAC61764.1	28	86.86	0.81
Smp_051400	Dynein DLC12	39	dynein light chain	NP_080832.1	3	10.4	0.74
Smp_021920	Paramyosin	35	mCG140437, isoform CRA_b	EDL10426.1	25	97	0.74
Smp_086330	Calponin	47	calponin-1 isoform X1	XP_011240690.1	7	21.2	0.70
Smp_068530	Syntenin	39	syntenin-2	NP_663510.1	8	32.24	0.64
Smp_045200	Sm22.6	32	Calm5 protein	AAI38692.1	4	22.6	0.55
Smp_085540	Myosin heavy chain	50	myosin-7	NP_542766.1	46	170.99	0.54
Smp_003990	TPI	64	TPI	AAC36016.1	9	28.1	0.50
	Dynein DLC13	45	dynein light chain	NP_080832.1	2	9.3	0.48
Smp_097800	Y box	65	Y box transcription factor	AAB94768.1	7	23.8	0.45
Smp_056970	GAPDH	74	GAPDH isoform 2	NP_032110.1	12	36.4	0.45
Smp_024110	Enolase	72	beta-enolase	NP_031959.1	15	47	0.44
Smp_009760	14-3-3 chain	64	14-3-3 zeta	BAA11751.1	8	28.4	0.44
Smp_072640	Stomatin	58	stomatin-like protein 2	NP_075720.1	10	39.53	0.44
Smp_042160	Aldolase	68	Aldolase C	AAH04802.1	10	35.4	0.42
	Mean	49.67					

^a^predicted by Bepipred (http://tools.immuneepitope.org/bcell/)

A different way of examining the vaccine potential of a protein is to determine the effects that evolutionary pressures might exert to alter the nucleotide sequence of the encoding gene. This can be estimated by analysing the rates of non-synonymous to synonymous substitutions (dN/dS) between orthologs from different schistosome species. Such a study was recently performed with the micro-exon (MEG) and venom allergen-like (VAL) genes of schistosomes [75]. The proteins they encode are associated with the glands and secretions of different life cycle stages [76–79], positioned at the parasite-host interface where they will be exposed to selection pressure from the immune system. The two classes of genes revealed significantly higher dN/dS values when compared with a set of control genes coding for secreted proteins, and for other proteins previously localized to the tegument. Analyses of paralog genes indicated that exposure of the protein to the definitive host immune system was indeed a determining factor leading to the higher dN/dS values. In addition, two other proteins (Sm29 and TSP-2) exposed at the tegument surface, and several lipid-processing proteins present in the worm vomitus displayed dN/dS values similar to those observed for MEGs and VAL genes. This provides further evidence of an additional selective pressure from the immune system on exposed proteins rather than a phenomenon specific for MEG and VAL genes. In complete contrast, almost all previously proposed vaccine candidates display very low rates of non-synonymous changes (Table 2). This is entirely consistent with their internal location and inaccessibility to immune effector responses in the live parasite. It also explains their strong immunogenicity when finally presented to the immune system upon release from the damaged or dead parasite. They have not been selected to be immunologically silent. This makes them the ideal agents to elicit a strong acquired response when administered multiple times with an adjuvant. Furthermore, when cercarial challenge is given only 10 to 15 days after the last boost, associated host responses to these reactive proteins will be maximal in the circulation, and so well placed to interfere non-specifically with an already fraught parasite migratory process.

**Table 2:** *dN/dS* measurements for genes encoding vaccine candidates

Vaccine candidate	Sma × Sha	Sma × Sja
Sm29	0.83	0.41
TSP-2	0.34 (0.65^a^)	0.12 (0.33^a^)
GST-28	0.32	0.16
SmTOR	0.3	0.2
Sjserpin	0.29	0.41
SjTGR	0.29	0.09
SjVLDL	0.27	0.11
Sm22.6	0.21	0.25
StoLP-2	0.2	0.05
Calpain	0.18	0.15
Sm21.7	0.18	0.53
SjCathepsin	0.15	0.13
GAPDH	0.14	0.06
SmRho	0.11	0.13
TPI	0.11	0.13
Sm14	0.11	0.06
Sm23	0.08	0.1
SOD	0.07	0.14
Aldolase	0.05	0.02
Sj22.7	0.04	0.004
Sm14-3-3	0.03	0.19
Myosin heavy chain	0.02	0.03
Paramyosin	0.01	0.04

Sma, S*chistosoma mansoni*; Sha, *S. haematobium*; Sja, *S. japonicum*

^a^Value for the portion of the gene encoding the exposed

hydrophilic loop utilised in vaccine trials

### What can protection experiments with heterologous antigens tell us?

Our hypothesis is that protection in the mouse model may be a bystander effect caused by high levels of circulating cytokines or the presence of activated T cells, macrophages and other leucocytes in the circulation, coincident with arrival of challenge larvae in the lungs. A corollary is that vaccination with irrelevant antigens should produce the same effect. The extensive experiments with live BCG are evidence for an “irrelevant” antigen effect on parasite maturation [73] but other heterologous proteins have been tested. Crude *Fasciola hepatica* extracts protected mice against schistosome challenge [80] with the major activity attributed to an abundant and immunogenic 12 kDa FABP [81]. Independently the *S. mansoni* homologue of this *Fasciola* protein was cloned, shown to elicit protection in mice and proposed as a dual *Fasciola/Schistosoma* vaccine [82, 83]. Does the cross-protection result from shared epitopes between the two proteins? The sequence homology is only 49 %, spread evenly throughout the polypeptide, and BepiPred does not identify stretches of amino acids in common that might serve as shared epitopes. This evolutionary distance is confirmed by a comparison with *S. bovis* FABP which has also been the subject of *Fasciola* cross-protection studies [84]. It differs from its *S. mansoni* equivalent at only two amino acid positions but is again only 49 % identical to the *F. hepatica* protein. Only epitope mapping of the respective fatty acid binding proteins with specific antisera can resolve this point.

Protection experiments have also been performed using heterologous proteases as adjuvants to boost protective responses to vaccine candidates such as GAPDH and 14-4-3 *via* a Th2-mediated immune response. It is remarkable that papain alone, from the plant *Carica papaya*, could induce a > 50 % reduction in worm burden when mice were challenged 14 days after a second administration of the enzyme subcutaneously [85]. The logic of this approach was that papain can activate basophils to secrete Th2-type cytokines in the absence of antigen-specific IgE [86]. These cells normally comprise ~1 % of circulating leucocytes and it would be pertinent to discover if they had any effect on circulating cytokines or pulmonary inflammation in papain-treated mice. In a similar way, functionally active *F. hepatica* cathepsin L1, which shares 47 % identity with its *S. mansoni* orthologue, is capable alone of inducing up to 50 % reduction in worm burden when injected 14 days before cercarial challenge [87]. When co-administered with schistosome proteins, a reduction in burden of up to 73 % may ensue. (Remember that this is 73 % of 32 %, i.e., 23 worms stopped *via* the immune response against 68 from natural causes, so still only a quarter.) A plausible alternative to an adjuvant role for these proteases, and the reactivity of *F. hepatica* FABP is that we are witnessing a bystander effect in the circulation impacting on the lungs, not specific acquired immunity.

## Do vaccine antigens identified in mice protect non-human primates?

A perplexing feature of the candidates identified in mouse vaccine trials is that they do not translate well to a permissive primate host like the baboon (*Papio anubis*). Note that since maturation in baboons can be > 80 % of penetrant cercariae, an acquired immune response has much more work to do than in a mouse to achieve a high level of protection. Nevertheless, > 80 % protection has been induced in primates like the baboon by multiple doses of the RA vaccine [17]. The > 80 % level of maturation means that only 20 out of 100 parasites die of “natural causes” during migration and a further 64 die due to immune effector mechanisms, the exact reverse of the mouse situation. The protection elicited in the baboon by the RA vaccine is also additive, in proportion to the number of exposures to attenuated cercariae, and a clear saw-tooth pattern of boost and decline in specific antibody production is observed with each successive vaccination [88]. The duration of protection has also been tested; it declines from 72 to 53 % when the interval between last boost and challenge is extended for three weeks to three months. Thus we contend that the baboon provides a robust test of vaccine potential. How well have vaccination experiments performed using the candidate antigens first trialled in mice?

A trial with SmGST28 produced contradictory results [89], depending on the adjuvant used with -26 and 38 % protection achieved. Another trial with myosin heavy chain (IrV5) gave 25 and 26 % protection in two different adjuvant formulations [90]. More recently, the tegument antigen Sm29 proved to be immunogenic but failed to induce protection (Kariuki, personal communication) while three anti-oxidant enzymes (two superoxide dismutases and glutathione peroxidase) in a DNA vaccine formulation all failed to reduce worm burden significantly, but did depress faecal egg excretion [91]. Only one vaccine candidate, Sm-p80 calpain has achieved a good level of protection in baboon: 48 % with a DNA construct [92], then 52–58 % with a recombinant protein [93] (there was a 4 week gap between last boost and challenge). Overall, it appears harder to elicit protection with single antigen formulations in baboons than in mice against a *S. mansoni* cercarial challenge: only calpain passes the primate test but persistence of protection induced does not yet appear to have been tested.

## Are *S. japonicum* vaccine experiments in mice subject to the same constraints?

In comparison with *S. mansoni* there is much less detailed information available about the mouse model infected with *S. japonicum*. However, the percentage maturation in naïve mice appears to be higher at ~50 % ([94]; Chinese strain), while that in hamsters (~53 %) is about the same [95], and in rabbits it is 53.5 % [96]. The pattern of schistosomulum migration and the site of elimination of non-maturing parasites are less well understood but an early study concluded that migration was entirely in the bloodstream, with passive transport between organs and active crawling through capillary barriers [97]. A more rapid migration of *S. japonicum* was noted with peak numbers in the lungs at 3–4 days and the first arrivals in the portal vein from 3.5 days. This qualitative pattern was later confirmed by mincing and incubation of tissues to recover migrating parasites [98]. The single quantitative autoradiographic tracking study reported that the skin was not a site of attrition after primary infection but that parasite elimination occurred after migration to the lungs and continued up to the liver stage [99]. The presence of large numbers of petechial haemorrhages on the lung surface has been recorded in several studies [97, 99, 100]; they are seldom observed in *S. mansoni*-infected hosts in spite of numerous schistosomula entering the alveoli. Most striking, petechiae have been recorded on other organs like the kidney, and on the walls of the stomach [97]. These observations confirm that migration occurs through systemic organs and that schistosomula arrive in the portal system *via* the capillary beds of splanchnic organs. Based on minimum recorded length, *S. japonicum* schistosomula are apparently one third larger than those of *S. mansoni* [97] so may cause greater vascular damage. In addition, given the > 50 % maturation rate, they may also have a greater capacity to re-enter the tissues to continue migrating.

Protection can be induced by exposure of mammalian hosts to RA cercariae of *S. japonicum*, often attenuated with UV light (reviewed in [101]). Furthermore, multiple vaccinations enhance that protection, compared to single vaccinations, as observed in mice, [102], pigs [103] and cattle [104]. The immunological basis of protection has been confirmed by passive transfer of serum from 5× vaccinated mice to naive recipients [91], exactly as with *S. mansoni* [50]. The most common immunization protocol for antigens is three immunizations, the first in complete Freund’s adjuvant, followed by two boosts with incomplete Freund’s adjuvant. Two weeks after immunization, mice are challenged with cercaria, usually 40, but sometimes as few as 20, due to the greater pathogenicity of *S. japonicum*. In contrast to the RA vaccine, antigens including the six most promising candidates selected by WHO, usually induced protection ranging from 20 to 40 %, if any (reviewed in [105]). Some researchers have reported that two antigens administered together can induce higher protection [106, 107], while others reported the opposite, compared to single antigens [108]. *S. japonicum* SWAP, presumably containing multiple candidates, has been reported to elicit > 40 % protection [109]. Some unexpected antigens also induced protection; for example, hypoxanthine-guanine phosphoribosyltransferase conferred over 40 % protection against challenge [110] and mitochondrial succinic dehydrogenase was also reported to induce significant protection [111]. The *S. japonicum* counterparts of surface proteins reported to elicit good protection against *S. mansoni*, (SjTSP2 [112], Sj29 [113]), were not as effective when used to vaccinate mice before challenge with *S. japonicum* cercariae. Finally, protection has been induced in mice against *S. japonicum* challenge using heterologous antigens as diverse as *Lumbricus terrestris* (earthworm; [114]) and *Trichinella spiralis* extracts [115]. Although the evidence is more fragmentary, we suggest that our bystander hypothesis is equally applicable to *S. japonicum* vaccine experiments in mice.

## Conclusions

In the title of this review we ask if there is a flaw in the mouse model. We have built a case that indeed there is, namely the fragility of the pulmonary capillaries. The consequence is that during lung transit in a naïve mouse, schistosomula burst into alveoli and have only a limited capability to continue migration thereafter; this is very much the dominant determinant of parasite maturation. There is good evidence that the RA vaccine makes pulmonary migration more difficult *via* a specific acquired response to surface and secreted antigens of the schistosomulum; additional worms are deflected but they are not harmed by the inflammatory responses. Conversely, our hypothesis is that for many of the proposed vaccine antigens the vaccination protocol increases the difficulty of pulmonary migration in a non-antigen-specific way. The kinetics of T cell production after vaccination with an antigen will likely mirror those after exposure to the RA vaccine. As the interval between last boost and challenge is usually short, activated cells will still be available in the circulation for recruitment to the lungs when the challenge parasites arrive. Note, such recruitment is not antigen-specific, but activation-specific. It is also likely that the level of pro-inflammatory cytokines in the circulation will be high, perhaps sufficient to modify the pulmonary vessels. This would be independent of the accessibility of the vaccine antigen in the live parasite. Our explanation also encompasses the protective effects of the various heterologous antigens - they are simply very immunoreactive.

### How can we probe the mouse model to test the assertions made in this review?

A major point of this overview of vaccine testing in the mouse was to provide a series of pointers for experiments to bring clarity to the situation. This is a plea to stop treating the mouse as a black-box test bed and make immunological measurements strictly in the parasitological context of challenge parasite migration and elimination. The onus is on vaccine researchers to show that their specific antigen model has a solid knowledge base in acquired immunity. Some key points are:

(i) What happens to the level of protection if the interval between the last boost and cercarial challenge is extended at least to five weeks and preferably longer? The very short interval of 10–15 days is a severe criticism of many antigen vaccine experiments.
(ii) What is the profile of activated T cells and of cytokines in the circulation after vaccination? Has it declined to background levels before cercarial challenge? The emphasis here is on circulation, not spleen or lymph nodes. Proliferation of peripheral blood mononuclear cells, cytokine production with and without antigen restimulation, detection of a DTH response by footpad or pinna swelling, are all appropriate assays. Key signatures would be IFNγ, TNFα or nitric oxide production, or the presence of activated monocytes/macrophages.
(iii) Does percutaneous cercarial challenge at 5 weeks or later elicit a detectable secondary response to the target antigen in the circulation of the antigen-vaccinated mice in the days immediately after challenge, measured as above? This is vital missing evidence that the living challenge larvae can trigger a recall response to vaccine antigens, especially internal ones.
(iv) Where are challenge parasites eliminated in antigen-vaccinated animals? Skin, lungs or later, we simply do not know. This was crucial to understanding how the RA vaccine operated. Although ^75^Se-Methionine is no longer commercially available, ^35^S Methionine and Cysteine provide an intensity of radioactive label that is still sufficient to allow detection of parasites in the skin and lungs by autoradiography so the question of elimination in those sites can be explored.
(v) Continuing the possibility of lung involvement, is there evidence for cell recruitment to the lungs after challenge that might interfere with migration in a non-antigen specific way? This is testable by broncho-alveolar lavage, and flow cytometric phenotyping of recovered populations.
(vi) Are there physiological or pharmacological interventions that might alter the migratory profile of schistosomula through the lung vasculature, independent of vaccination experiments? Does i.v. administration of pro-inflammatory cytokines such as IFNγ or TNFα diminish schistosome maturation as implied by the RA vaccine? Alternatively, can we emulate the protection achieved with the RA vaccine using pharmacological interventions? Molecules that disrupt pulmonary hemodynamics could in theory modify the barrier to parasite migration. In this context, would nitric oxide-generating molecules, by promoting vasodilation, significantly alter vascular tonus in the lungs? This would be difficult to test due to the short-lived nature of these molecules; perhaps their effects could be sustained by the use of phosphodiesterase inhibitors. Other molecules capable of altering lung fluid homeostasis (e.g., leukotriene D4, platelet-activating factor, and thromboxane A2 mimetics) could represent potential drugs to be tested.

A recent issue of Frontiers in Immunology was entitled “The Schistosomiasis Vaccine - It is Time to Stand up”. We doubt on present evidence that many claims of efficacy are plausible. The reservations we raise about the mouse as a test-bed for schistosome vaccine antigens require thorough scrutiny. It is our contention that due to the poor level of maturation of *S. mansoni* parasites, almost any laboratory host would be a better option (only the rat is worse). The possibility of bystander effects drastically altering migration and maturation should diminish in hosts where a greater proportion of penetrants mature. On that basis, the hamster would be a better choice for large scale tests with *S. mansoni* and the rabbit with *S. japonicum*. The baboon provides the ultimate choice as a permissive primate. Its body mass, typically 6–10 kg, coupled with a high percentage maturation of penetrant cercariae, point to a robust pulmonary blood/air barrier keeping accidental parasite loss to the alveoli to a minimum. The baboon’s ability to tolerate a large cercarial challenge without developing severe of lethal pathology and its phylogenetic proximity to *Homo sapiens* are further positive attributes. We suggest that the results of antigen trials in baboons should be in place before the expensive scale-up to human trials is ever contemplated.

## Additional file

Additional file 1:
**Spreadsheet calculations documenting the predicted migration profile of schistosomula when the probablility of getting stuck in the lungs on each circuit of the vascular system is modified.** **a** Cercarial challenge, maturation in the naive mouse = 32 %. **b** Intravenous injection of day 7 schistosomula to the pulmonary vasculature, maturation = 50 %. **c** Cercarial challenge of an antigen-vaccinated mouse, 40 % protection. **d** Ditto, 70 % protection. (XLSX 31.2 kb)

## Abbreviations

i.v.: intravenous
IFNγ: interferon gamma
TNFα: tumour necrosis factor alpha
